# Inequalities in colorectal cancer screening participation in the first round of the national screening programme in England

**DOI:** 10.1038/sj.bjc.6605392

**Published:** 2009-12-03

**Authors:** C von Wagner, A Good, D Wright, B Rachet, A Obichere, S Bloom, J Wardle

**Affiliations:** 1 Department of Epidemiology and Public Health, Cancer Research UK Health Behaviour Research Centre, University College London, Gower Street, London WC1E 6BT, UK; 2 Department of Clinical Biochemistry, Northwick Park Hospital, North West London Hospitals NHS Trust, Watford Rd., London, Harrow, UK; 3 Department of Epidemiology and Population Health, Cancer Research UK Cancer Survival Group, Non-Communicable Disease Epidemiology Unit, London School of Hygiene and Tropical Medicine, Keppel Street, London, UK; 4 Department of Gastroenterology, University College London Hospital, Maple House, 25 Grafton Way, London, UK

**Keywords:** colorectal cancer screening, socio-economic status, ethnicity, self-reported health, health inequality

## Abstract

**Background:**

Introduction of organised, population-based, colorectal cancer screening in the United Kingdom using the faecal occult blood test (FOBT) has the potential to reduce overall colorectal cancer mortality. However, socio-economic variation in screening participation could exacerbate existing inequalities in mortality.

**Methods:**

This study examined FOBT uptake rates in London, England in relation to area-level socio-economic deprivation over the first 30 months of the programme during which 401 197 individuals were sent an FOBT kit. Uptake was defined as return of a completed test kit within 3 months. Area-level deprivation in each postcode sector was indexed with the Townsend Material Deprivation Index. Analyses controlled for area-level household mobility, ethnic diversity and poor health, each of which was associated with lower return rates.

**Results:**

The results showed a strong socio-economic gradient in FOBT uptake, which declined from 49% in the least deprived quintile of postcodes to 38% in the middle quintile and 32% in the most deprived quintile. Variation in socio-economic deprivation between sectors accounted for 62% of the variance in return rates, with little attenuation as a result of controlling for ethnic diversity, household mobility or health status.

**Conclusion:**

These results highlight the need to understand the causes of socio-economic gradients in screening participation and address barriers that could otherwise increase disparities in colorectal cancer survival.

Reducing health inequalities through equality of access to health care is a cornerstone of the UK Government's health policy, and has been re-emphasised in the Department of Health (2007). However, associations between socio-economic status (SES) and health have persisted through major changes in social conditions and provision of medical care, and it has been argued that innovations in health technology may widen inequalities if people with more knowledge, money or power are better able to harness the beneficial effects (Link *et al*, 1998; Baker and Middleton, 2003; Coleman *et al*, 2004).

The UK Breast and Cervical Cancer Screening Programmes were adopted more enthusiastically in more affluent areas (Gatrell *et al*, 1998; Webb *et al*, 2004; Dailey *et al*, 2007), and at least in the case of cervical cancer, this has had a measurable impact on disparities in mortality (Link *et al*, 1998). As part of the National Health Service (NHS), cancer screening in the United Kingdom incurs no direct financial costs, so affordability cannot explain variation in uptake. However, both breast and cervical screening require attendance for a clinic appointment, and therefore factors such as pressures of time, transport problems or discomfort in interacting with medical services and health professionals, could lie behind SES differences in participation.

Whether the same SES differential will be seen for the latest addition to the UK Cancer Screening Programmes, biennial bowel cancer screening using the faecal occult blood test (FOBT), is an important question. In the Bowel Cancer Screening Programme (BCSP), all 60–69-year olds are sent an invitation to participate around a week before the test kit is mailed to the home address. The kit is accompanied by step-by-step instructions on how to complete the test at home and a hygienic freepost envelope is provided to return the samples to the laboratory for processing. Individuals are informed of the result and advised if further tests are needed (repeat FOBT or colonoscopy) within 2 weeks.

Faecal occult blood test has the potential to reduce colorectal cancer mortality by 16% (Hewitson *et al*, 2008) but SES differences in participation could increase existing differentials in survival (Mitry *et al*, 2008). The BCSP has some features that ought to minimise social inequalities: it incurs no direct or indirect financial cost to the individual, no time off work is required, and the test is delivered direct to the home, is self-administered and returned in a ‘freepost’ envelope. This did not prevent SES differences in the UK trial of FOBT screening (Whynes *et al*, 2003) or the second round of the UK colorectal screening pilot (Weller *et al*, 2007). However, as inequalities in participation in the cervical screening programme have narrowed over time (Baker and Middleton, 2003), it is possible that this will occur in the context of the BCSP.

This study assessed FOBT participation in relation to neighbourhood socio-economic characteristics in the London area in the first 30 months of the national roll out of the BCSP. Data were based on test kit return rates for 401 197 individuals aged 60–69, resident in 808 postcode sectors, who were sent FOBT kits between October 2006 and January 2009.

## Materials and methods

### Implementation of the screening programme in London

In 2006, the Department of Clinical Biochemistry at Northwick Park and St Marks Hospitals became the London ‘Hub’ for the BCSP, responsible for sending out and analysing FOBT kits, and supported by six screening centres carrying out follow-up investigations. The analyses reported here are based on 401 197 first invitations sent out by the Hub across 808 postcode sectors between October 2006 (when the programme went ‘live’) and January 2009. Kits were recorded as returned if they were received by the screening centre within 3 months.

Owing to the way the programme is organised, not all eligible individuals would have been invited during the first 30 months of the programme. We therefore divided the number of invitations sent to a postcode sector by the number of people in the age range living in that sector (data from April 2009) to estimate screening coverage. On average, 497 invitations were sent per postcode sector, giving an average of 68% coverage (s.d.=17.0) during the study period.

### Area-level deprivation

Postcode sectors are areas defined by the first inward digit of the postcode and contain an average of 3000 addresses. Socio-economic deprivation for each postcode sector was indexed with the Townsend Material Deprivation Index (Townsend *et al*, 1988). This is based on four area-level indicators from the most recent Census: levels of unemployment among those who are economically active, owner-occupancy (% of homes owned by the occupier), car ownership (% with a car) and home overcrowding (% with no communal living space or less than one bedroom per single adult, couple or pair of children) (Mackenzie *et al*, 1998). Higher Townsend scores indicate greater deprivation.

### Other area-level measures

We used 2001 Census data (Office for National Statistics, 2001, 2004) to include the following additional factors in the analyses: the proportion of people in each postcode sector belonging to non-white ethnic groups (i.e. all ethnic groups other than ‘white British’, ‘white Irish’ and ‘white Other’), levels of reported poor health (% of people describing their health as ‘not good’) and household mobility (% of people who had reported moving out of an area within 12 months before the 2001 Census). Household mobility may cause patient lists to be inflated by records of patients no longer residing in the area.

### Analyses

Analyses were carried out using SPSS, version 14.0 (SPSS, Inc., Chicago, IL, USA). Postcode sectors were grouped into quintiles of Townsend scores to examine the association between area-level deprivation and uptake using the *χ*^2^ test for linear trend. Ethnic diversity and area mobility variables were transformed to correct for skewed distributions: [log_10_(ethnic diversity+6); log_10_(area mobility+1)]. Linear regression analysis using SPSS Complex Samples was used to examine the independent effect of deprivation after controlling for other area-level factors.

As the percentage of the eligible population who had been sent a kit during the first 30 months of the programme varied from 2% to 97% across the 808 postcodes, and there was a marginally significant trend for variation in coverage across deprivation quintiles (*P*=0.07), data were weighted by coverage in further analyses.

## Results

**Figure 1:**
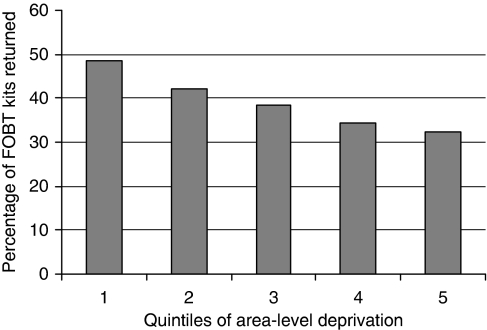
FOBT return by quintile of area-based socio-economic deprivation (*N*=401 197).

The percentage of individuals returning kits increased from 32% in the most deprived quintile, through 34%, 38% and 42%, to 49% in the least deprived quintile (see Figure 1). An important observation here is that the results indicate a gradient across quintiles (linear trend: *P*<0.001) rather than a sudden ‘drop-off’ in uptake for the most deprived quintile. Univariate regression analysis (see Table 1) demonstrated that area deprivation accounted for 62% of the variance in response rates across sectors.

Test kit return was also lower for postcode sectors with higher ethnic diversity, area mobility or poor health (all *P*s<0.001), characteristics that were all associated with area deprivation. Regression analyses were used to examine the extent to which deprivation was associated with screening uptake independently of ethnic diversity, poor health or mobility. The results (see Table 2) showed that associations between each of these neighbourhood characteristics and uptake were substantially reduced after including deprivation in the model. Meanwhile the *β* coefficient for deprivation was reduced only slightly by the addition of any of these predictors and was actually increased by controlling for poor health.

## Discussion

These analyses demonstrate a strong SES differential in uptake of the FOBT-based BCSP in London. Residents in the most affluent areas were significantly more likely to return the test kits than those in slightly less affluent (though nonetheless still well-to-do) areas, and this pattern continued linearly through the quintiles of deprivation. Controlling for area mobility as an indicator of unreliable address records made no difference to the gradient. Uptake in the most affluent quintile was 50% higher than in the most deprived quintile, showing as much of a gradient as was seen in the early days of the breast or cervical screening programmes (Majeed *et al*, 1995; Baker and Middleton, 2003). While longitudinal analysis of cervical screening uptake showed a narrowing of the gradient over time (Baker and Middleton, 2003), and this may also turn out to be true for the bowel screening programme, there is a need for immediate action to reduce inequity in delivery. The magnitude of the deprivation effect is particularly worrying given that the greater economic and socio-cultural mix in London might have been expected to dilute the association between indicators of area-level deprivation and screening uptake. Other area-level indicators that are known to be associated with deprivation and are of particular relevance in London, such as ethnic diversity of area mobility, did not explain the socio-economic variation. Some of the shortcomings associated with our measures (discussed in more detail below) should be considered when interpreting this finding. The measure of ethnic diversity was relatively simplistic and data regarding area mobility would have benefited from a more up-to-date source, but notwithstanding these limitations, it remains an important finding of the present analysis that there was a strong and linear gradient of deprivation on uptake in bowel cancer screening in London that could not be explained by ethnic diversity or area mobility. Consequently, strategies such as translating the materials to other languages or addressing culturally specific attitudes to screening, important as these are in their own right, may only achieve a small impact on the deprivation effect.

The above finding should not detract from the observation that ethnic diversity contributed independently to lower uptake, similar to the effect observed in the English pilot study of the BCSP, which found lower uptake in areas with higher proportions of residents from the Indian subcontinent (Whynes *et al*, 2003). In this ethnic group, there was evidence that people had more difficulty in completing the test and were more likely to perceive it as unhygienic, embarrassing and distasteful, pointing to some possible avenues for intervention.

The finding that areas with a larger proportion of the population reporting poor health achieved *higher* uptake accords with individual-level studies identifying perception of good health and absence of symptoms as barriers to early detection (Ogedegbe *et al*, 2005) including uptake of FOBT (Ioannou *et al*, 2003). However area-based measures are less easily interpreted because of the possibility that high concentrations of individuals with poor health may influence the provision or quality of health-care services (for example, in the form of health promotion efforts). Nevertheless, the fact that controlling for poor health *increased* the observed SES gradient highlights the need to emphasise to the public that screening is for everyone, and not just people with symptoms (Brotherstone *et al*, 2006). This may have implications for the information materials that accompany the test kit.

It has been argued that innovations in health technology can widen inequalities if people with more knowledge, money or power are better able to harness the beneficial effects (Link *et al*, 1998; Baker and Middleton, 2003; Coleman *et al*, 2004). While it is not immediately obvious that ‘knowledge, money or power’ explain SES differences in performance of a comparatively simple home test that has no associated cost and requires no social interaction, the fact that the gradient matches that observed for other forms of screening and other health behaviours, highlights the importance of understanding broader influences on engagement with preventive health behaviours, particularly in relation to cancer prevention.

In the meantime, there is an urgent need to identify midstream or downstream strategies that can ameliorate inequalities in participation in the BCSP. Explanations put forward for social differentials in health behaviours often focus specifically on the lowest SES group (e.g. severe difficulties in health literacy), and although addressing these may not affect the whole gradient, they could contribute to better health outcomes in the most disadvantaged sector (von Wagner *et al*, 2009). One potential explanatory factor that has been shown to have a graded association with SES is the perceived value of early detection of cancer (Wardle *et al*, 2004). Fatalism about cancer has been shown to be associated with SES (Powe and Finnie, 2003; Wardle *et al*, 2004) and could contribute to reluctance to engage with screening if early detection is perceived as creating a longer period of suffering before inevitable death. Strategies to promote a more positive view of cancer outcomes could have a role here. Another factor is future orientation. There is some evidence that SES is related to the relative value attached to future (*vs* present) gains and losses (Wardle and Steptoe, 2003). If the perceived benefit of better survival a long way ahead is more highly discounted by lower SES groups, it could be insufficient to offset the unpleasantness of collecting faecal samples in the short term. There is no doubt that some people find the idea of completing this test at least unpleasant if not ‘disgusting’ (Chapple *et al*, 2008), particularly because touching faecal material is taboo in all societies. The numbers of toilets or private places in the home to store the samples could have a role in compliance; which could be related to SES, but interestingly, there is also some evidence for a social gradient in the ‘disgust’ response (Rozin *et al*, 2000). Modifying the sample collection procedure by, for example, introducing alternative testing modalities requiring fewer samples, could contribute to reducing the SES gradient in test participation.

### Limitations

There were limitations to this study. Area-based measures of socio-economic deprivation may be out-of-date because they are usually based on census data collected at 10-yearly intervals, but this should serve only to underestimate the deprivation effect. There can also be large variations in levels of personal deprivation within an area; which are not identified with area-based measures (Drever, 1995) making it impossible to draw definite conclusions about associations between uptake and individual SES. The use of smaller geographical units (e.g. Lower Super Output Areas) as well as commercial geo-demographic classification systems, such as ACORN or MOSAIC that supplement census level data with up-to-date demographic and lifestyle data, would give a finer resolution. We also lacked data on sex or age, which might have influenced or interacted with factors in our analysis such as perceived health status, but these require individual-level data that were not available for these analyses. Variability in coverage may have compromised the extent to which we could generalize the current findings although we did control for coverage in our analysis. It was also reassuring to find that excluding postcode sectors with less than 50% coverage (*N*=201) did not alter the effect of area-based socio-economic deprivation. Finally, this analysis was restricted to test kits sent out in London, which, as noted above, has a very heterogeneous population. Future studies addressing inequalities in bowel cancer screening will benefit from data from the ‘hubs’ delivering the programme throughout the country, which will provide information about additional issues such as differences between urban and rural areas.

## Conclusions

This analysis has demonstrated a striking and linear association between area-level deprivation and the proportion of individuals returning an FOBT kit as part of the UK BCSP; which is not explained by differences in ethnic diversity, area mobility or health status. It does not provide explanations, but does emphasise the urgency of constructing more comprehensive multi-level explanatory models. In the meantime, the fact that area-level characteristics predict participation provides an opportunity to address midstream factors that maintain disparities in uptake through tailored educational and instructional materials to offset some of the negative effects of deprivation.

## Figures and Tables

**Table 1:** Univariate predictors of area-level uptake of FOBT

	***B***	***b***	**95% CI**	***R* ^2^**
Deprivation	−0.78^**^	−1.74	−1.849, −1.636	**0.62**
Ethnic diversity^a^	−0.62^**^	−24.49	−26.774, −22.205	**0.38**
Area mobility^a^	−0.36^**^	−23.55	−28.691, −18.417	**0.13**
Poor health	−0.45^**^	−1.903	−2.234, −1.572	**0.21**

Abbreviation: FOBT=faecal occult blood test.

a Transformed variables. ^**^Significant at *P*<0.001. Bold values are effects size (*R*^2^) – the same level of significance as shown for the unstandardised regression coefficient (*B*) applies for these and thus does not need to be added to the bold values.

**Table 2:** Predicting area level uptake of FOBT: multivariate models weighted by coverage

	***B***	***B***	**95% CI**
*Model 1*			
Deprivation	−0.64^**^	−1.42	−1.541, −1.304
Ethnic diversity^a^	−0.25^**^	−9.98	−11.918, −8.032
*R*^2^	**0.66**		
			
*Model 2*			
Deprivation	−0.77^**^	−1.71	−1.826, −1.587
Area mobility^a^	0.03	2.14	−6.782, 1.606
*R*^2^	**0.62**		
			
*Model 3*			
Deprivation	−0.86^**^	−1.91	−2.068, −1.757
Poor health	0.12^*^	0.49	0.147, 0.825
*R*^2^	**0.63**		
			
*Model 4: full multivariate model*			
Deprivation	−0.71^**^	−1.57	−1.799, −1.367
Ethnic diversity^a^	−0.28^**^	−10.97	−12.978, −8.966
Area mobility^a^	−0.02	−1.48	−6.570, 3.256
Poor health	0.14^*^	0.61	0.191, 0.980
*R*^2^	**0.68**		
			

Abbreviation: FOBT=faecal occult blood test.

a Transformed variables.

^**^Significant at *P*<0.001, ^*^significant at *P*<0.05. Bold values are effects size (*R*^2^) – the same level of significance as shown for the unstandardised regression coefficient (*B*) applies for these and thus does not need to be added to the bold values.

## References

[bib1] Baker D, Middleton E (2003) Cervical screening and health inequality in England in the 1990s. J Epidemiol Community Health 57: 417–423 12775786 10.1136/jech.57.6.417 PMC1732483

[bib2] Brotherstone H, Miles A, Robb KA, Atkin W, Wardle J (2006) The impact of illustrations on public understanding of the aim of cancer screening. Patient Educ Couns 63: 328–335 17011155 10.1016/j.pec.2006.03.016

[bib3] Coleman MP, Rachet B, Woods LM, Mitry E, Riga M, Cooper N, Quinn MJ, Brenner H, Esteve J (2004) Trends and socioeconomic inequalities in cancer survival in England and Wales up to 2001. Br J Cancer 90: 1367–1373 15054456 10.1038/sj.bjc.6601696 PMC2409687

[bib4] Chapple A, Ziebland S, Hewitson P, McPherson A (2008) What affects the uptake of screening for bowel cancer using the faecal occult blood test (FOBt): a qualitative study. Soc Sci Med 66: 2425–2435 18358581 10.1016/j.socscimed.2008.02.009

[bib5] Dailey AB, Kasl SV, Holford TR, Calvocoressi L, Jones BA (2007) Neighborhood-level socioeconomic predictors of nonadherence to mammography screening guidelines. Cancer Epidemiol Biomarkers Prev 16: 2293–2303 18006918 10.1158/1055-9965.EPI-06-1076

[bib6] Drever F (1995) Mortality in regions and local authority districts in the 1990s: exploring the relationship with deprivation. Popul Trends 86: 19–26 8745102

[bib7] Department of Health (2007) NHS *Cancer Reform Strategy*. Department of Health: London

[bib8] Gatrell A, Garnett S, Rigby J, Maddocks A, Kirwan M (1998) Uptake of screening for breast cancer in South Lancashire. Public Health 112: 297–301 9807924 10.1038/sj.ph.1900492

[bib9] Hewitson P, Glasziou P, Watson E, Towler B, Irwig L (2008) Cochrane systematic review of colorectal cancer screening using the fecal occult blood test (hemoccult): an update. Am J Gastroenterol 103: 1541–1549 18479499 10.1111/j.1572-0241.2008.01875.x

[bib10] Ioannou GN, Chapko MK, Dominitz JA (2003) Predictors of colorectal cancer screening participation in the United States. Am J Gastroenterol 98: 2082–2091 14499792 10.1111/j.1572-0241.2003.07574.x

[bib11] Link BG, Northridge ME, Phelan JC, Ganz ML (1998) Social epidemiology and the fundamental cause concept: on the structuring of effective cancer screens by socioeconomic status. Milbank Q 76: 375–402 9738168 10.1111/1468-0009.00096 PMC2751089

[bib12] Mackenzie IF, Nelder R, Maconachie M, Radford G (1998) ‘My ward is more deprived than yours’. J Public Health 20: 186–190 10.1093/oxfordjournals.pubmed.a024741 9675738

[bib13] Majeed FA, Cook DG, Given-Wilson R, Vecchi P, Poloniecki J (1995) Do general practitioners influence the uptake of breast cancer screening? J Med Screen 2: 119–124 8536178 10.1177/096914139500200301

[bib14] Mitry E, Rachet B, Quinn MJ, Cooper N, Coleman MP (2008) Survival from cancer of the colon in England and Wales up to 2001. Br J Cancer 99: S26–S29 18813251 10.1038/sj.bjc.6604578 PMC2557549

[bib15] Office for National Statistics (2001) Census 2001: Key Statistics for postcode sectors in England and Wales. Crown Copyright: London

[bib16] Office for National Statistics (2004) Census 2001: Key Statistics for postcode sectors in England and Wales: supplementary files and corrections/CSV version 1 August 2004. Crown Copyright: London

[bib17] Ogedegbe G, Cassells AN, Robinson CM, DuHamel K, Tobin JN, Sox CH, Dietrich AJ (2005) Perceptions of barriers and facilitators of cancer early detection among low-income minority women in community health centers. J Natl Med Assoc 97: 162–170 15712779 PMC2568778

[bib18] Powe BD, Finnie R (2003) Cancer fatalism: the state of the science. Cancer Nurs 26: 454–465 15022977 10.1097/00002820-200312000-00005

[bib19] Rozin P, Haidt J, McCauley CR (2000) Disgust. In Handbook of Emotions, Lewis MJ, Haviland-Jones JM (eds), pp 637–653. Guildford Press: New York

[bib20] Townsend P, Phillimore P, Beattle A (1988) Health and Deprivation: Inequality and the North. London: Croom Helm

[bib21] von Wagner C, Semmler C, Good A, Wardle J (2009) Health literacy and self-efficacy for participating in colorectal cancer screening: the role of information processing. Patient Educ Couns 75(3): 352–357 19386461 10.1016/j.pec.2009.03.015

[bib22] Wardle J, McCaffery K, Nadel M, Atkin W (2004) Socioeconomic differences in cancer screening participation: comparing cognitive and psychosocial explanations. Soc Sci Med 59: 249–261 15110417 10.1016/j.socscimed.2003.10.030

[bib23] Wardle J, Steptoe A (2003) Socioeconomic differences in attitudes and beliefs about healthy lifestyles. J Epidemiol Community Health 57: 440–443 12775791 10.1136/jech.57.6.440 PMC1732468

[bib24] Webb R, Richardson J, Esmail A, Pickles A (2004) Uptake for cervical screening by ethnicity and place-of-birth: a population-based cross-sectional study. J Public Health 26: 293–296 10.1093/pubmed/fdh128 15454600

[bib25] Weller D, Coleman D, Robertson R, Butler P, Melia J, Campbell C, Parker R, Patnick J, Moss S (2007) The UK colorectal cancer screening pilot: results of the second round of screening in England. Br J Cancer 97: 1601–1605 18026197 10.1038/sj.bjc.6604089 PMC2360273

[bib26] Whynes DK, Frew EJ, Manghan CM, Scholefield JH, Hardcastle JD (2003) Colorectal cancer, screening and survival: the influence of socio-economic deprivation. Public Health 117: 389–395 14522153 10.1016/S0033-3506(03)00146-X

